# Supplementary material for: Glutathione and a Pool of Metabolites Partly Related to Oxidative Stress Are Associated with Low and High Myopia in an Altered Bioenergetic Environment
Source: Antioxidants (Basel). 2024 Apr 27;13(5):539. doi: 10.3390/antiox13050539 (PMC11117864; doi:10.3390/antiox13050539)
Supplement: Supplementary file 1 [file antioxidants-13-00539-s001.zip › antioxidants-2964807-supplementary.pdf]

Table S1. List of the chemical shifts used to make metabolite assignments in aqueous humor ^{1}H NMR spectra. The preoperative procedure involving the use of antibiotic eye drops (Oftalmowell®) conditioned the presence of two metabolites (*).

ppm	ID	Formula	PubChem Compound	CAS Registry
0,878	Valerate	C5H10O2	7991	109-52-4
0,894	Butyrate	C4H8O2	264	107-92-6
0,91	2-Hydroxybutyrate	C4H8O3	11266	565-70-8
0,949	Leucine	C6H13NO2	6106	61-90-5
0,97	2-Aminobutyrate	C4H9NO2	80283	1492-24-6
1,002	Isoleucine	C6H13NO2	6306	73-32-5
1,034	Valine	C5H11NO2	6287	72-18-4
1,061	3-Hydroxyisobutyrate	C4H8O3	87	2068-83-9
1,067	Isobutyrate	C4H8O2	6590	79-31-2
1,14	Propylene glicol*	C3H8O2	1030	57-55-6
1,169	Isopropanol	C3H8O	3776	67-63-0
1,179	Ethanol*	C2H6O	702	64-17-5
1,198	3-Hydroxybutyrate	C4H8O3	92135	625-72-9
1,324	Lactate	C3H6O3	107689	79-33-4
1,428	2-Phenylpropionate	C9H10O2	10296	492-37-5
1,471	Alanine	C3H7NO2	5950	56-41-7
1,63	Adipate	C6H10O4	196	124-04-9
1,715	Lysine	C6H14N2O2	5962	56-87-1
1,912	Acetate	C2H4O2	176	64-19-7
1,923	N-Acetyltyrosine	C11H13NO4	68310	537-55-3
2,229	Acetone	C3H6O	180	67-64-1
2,287	4-Aminobutyrate	C4H9NO2	119	56-12-2
2,309	Glutamate	C5H9NO4	33032	56-86-0
2,367	Pyruvate	C3H4O3	1060	127-17-3
2,398	Succinate	C4H6O4	1110	110-15-6
2,46	Glutamine	C5H10N2O3	5961	56-85-9
2,528	Citrate	C6H8O7	311	77-92-9
2,578	Glutathione	C10H17N3O6S	124886	70-18-8
2,609	Methylamine	CH5N	6329	74-89-5
2,722	Dimethylamine	C2H7N	674	124-40-3
2,73	Sarcosine	C3H7NO2	1088	107-97-1
2,896	Trimethylamine	C3H9N	1146	75-50-3
3,032	Creatine phosphate	C4H10N3O5P	9548602	67-07-2
3,036	Creatine	C4H9N3O2	586	57-00-1
3,04	Creatinine	C4H7N3O	588	60-27-5
3,149	Ethanolamine	C2H7NO	700	141-43-5
3,149	N-Nitrosodimethylamine	C2H6N2O	6124	62-75-9

3,187	O-Acetylcarnitine	C9H18NO4	18230	3040-38-8
3,193	Choline	C5H14NO	305	62-49-7
3,196	O-Acetylcholine	C7H16NO2	187	51-84-3
3,21	sn-Glycero-3-phosphocholine	C8H21NO6P	439285	28319-77-9
3,218	O-Phosphocholine	C5H15NO4P	1014	107-73-3
3,242	Taurine	C2H7NO3S	1123	107-35-7
3,257	Trimethylamine N-oxide	C3H9NO	1145	1184-78-7
3,281	myo-Inositol	C6H12O6	892	87-89-8
3,29	1,7-Dimethylxanthine	C7H8N4O2	4687	611-59-6
3,359	Methanol	CH4O	887	67-56-1
3,551	Glycine	C2H5NO2	750	56-40-6
4,017	Ascorbate	C6H8O6	5785	50-81-7
4,251	Threonine	C4H9NO3	6288	72-19-5
5,232	Glucose	C6H12O6	5793	50-99-7
7,007	τ-Methylhistidine	C7H11N3O2	92105	332-80-9
7,04	π-Methylhistidine	C7H11N3O2	64969	368-16-1
7,189	Tyrosine	C9H11NO3	6057	60-18-4
7,215	Tyramine	C8H11NO	5610	51-67-2
7,427	Phenylalanine	C9H11NO2	6140	63-91-2
7,53	Tryptophan	C11H12N2O2	6305	73-22-3
7,829	Histidine	C6H9N3O2	6274	71-00-1
8,445	Formate	CH2O2	284	64-18-6

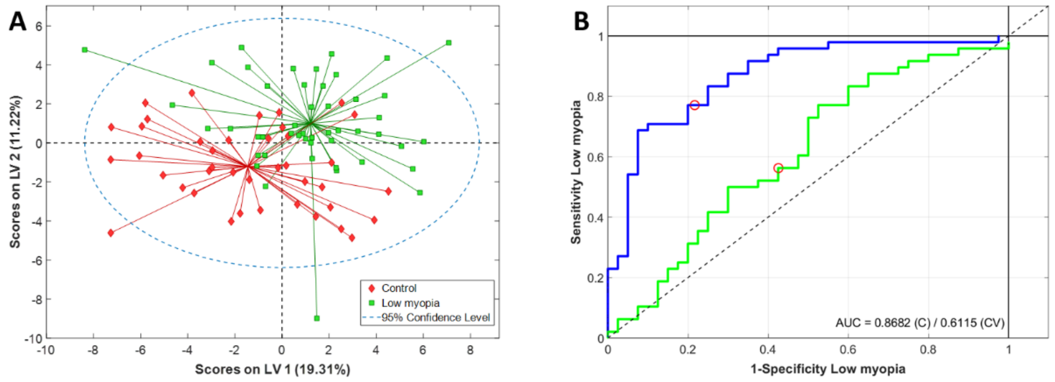

Figure S1. (A) Score plot of the PLS-DA of the metabolome for the discrimination between the control group (red diamonds) and LM group (green squares). (B) Receiver-operating curve (ROC) analysis for discrimination between the control group and HM samples based on our PLS-DA model.

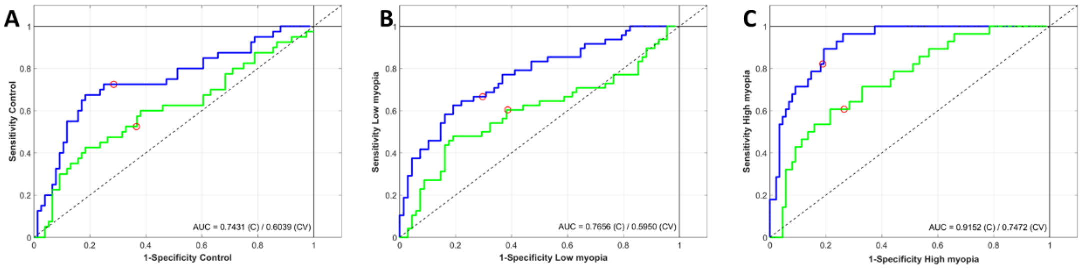

Figure S2: Receiver-operating curve (ROC) analysis for the classification of each individual group: (A) control group, (B) LM group, and (C) HM group based on our PLS-DA model.

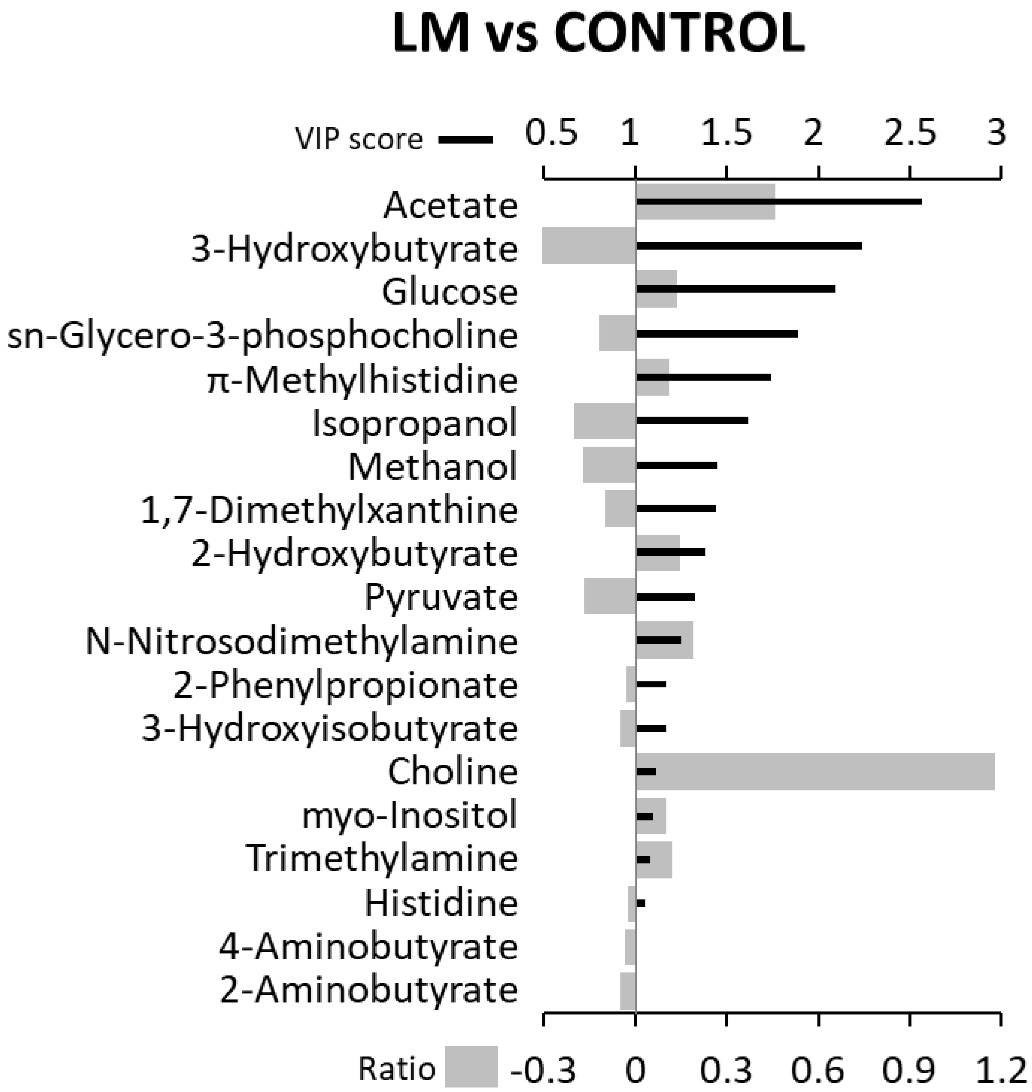

Figure S3: VIP score and relative fold change bar plot of the model for discriminating between the LM and control groups.

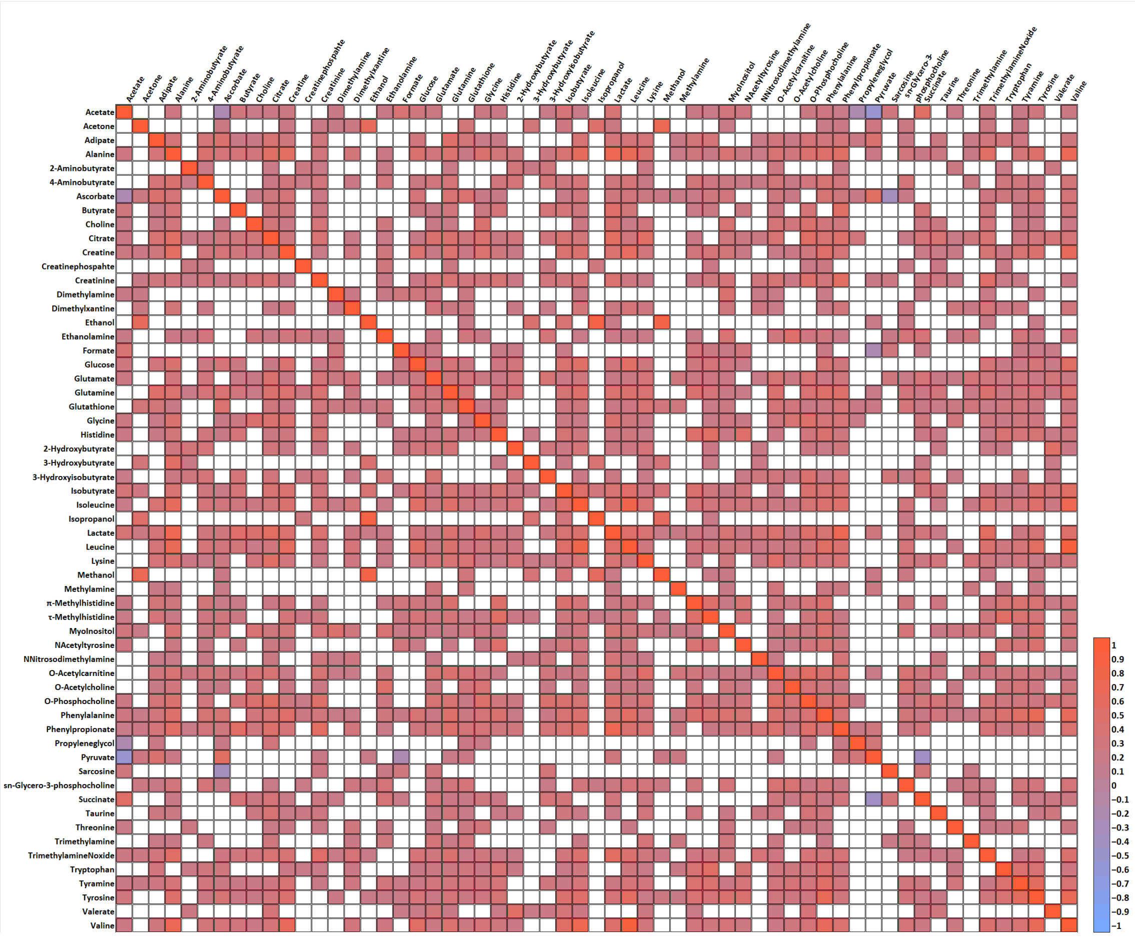

Figure S4: Spearman's rank correlation coefficient of the strength of linear relationships between AH metabolites (* $p < 0.05$).

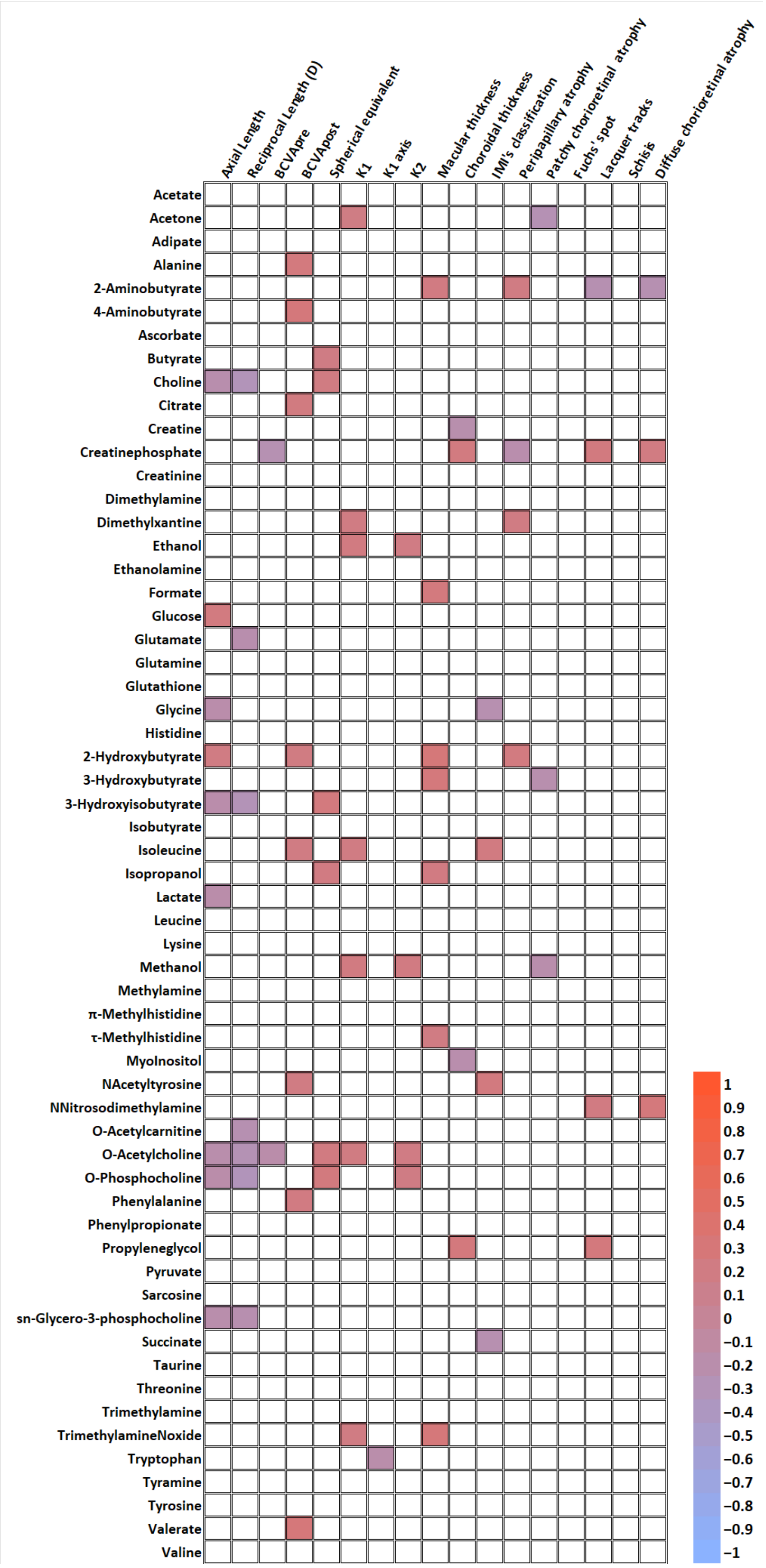

Figure S5: Spearman's rank corre-lation coefficient of the strength of linear relationships between paired clinical data and AH me-tabolites (* $p < 0.05$).
